# Detection of human cytomegalovirus cell‐free DNA in pregnant women with symptomatically infected fetuses: proof‐of‐concept study

**DOI:** 10.1002/uog.29199

**Published:** 2025-03-03

**Authors:** B. H. W. Faas, T. Meuleman, G. Astuti, A. Reuss, K. Stol, E. A. Sistermans, J. Linthorst, E. van Leeuwen, J. Rahamat‐Langendoen, F. A. Wilmink

**Affiliations:** ^1^ Department of Human Genetics Radboud university medical center Nijmegen Nijmegen The Netherlands; ^2^ Department of Obstetrics and Gynecology Radboud university medical center Nijmegen Nijmegen The Netherlands; ^3^ Department of Pediatrics, Amalia Children's Hospital Radboud university medical center Nijmegen Nijmegen The Netherlands; ^4^ Department of Human Genetics, Amsterdam UMC location Vrije Universiteit Amsterdam Amsterdam The Netherlands; ^5^ Amsterdam Reproduction & Development research institute, Amsterdam UMC Amsterdam The Netherlands; ^6^ Department of Obstetrics Amsterdam UMC location University of Amsterdam Amsterdam The Netherlands; ^7^ Department of Medical Microbiology Radboud university medical center Nijmegen Nijmegen The Netherlands

**Keywords:** cell‐free DNA, cytomegalovirus, non‐invasive prenatal testing, screening

## Abstract

**Objective:**

To evaluate the presence and levels of cytomegalovirus (CMV) cell‐free DNA (cfDNA) fragments in women pregnant with a fetus with symptomatic congenital CMV (cCMV).

**Methods:**

The study comprised nine women whose fetuses were diagnosed with cCMV between June 2019 and July 2024 at 20 + 4 to 34 + 1 weeks' gestation (*n* = 8) or neonatally (*n* = 1) after primary or non‐primary maternal infection. In eight women, cfDNA sequencing data from a single timepoint were analyzed, either retrospectively, on data generated from 11–13 weeks' gestation (*n* = 5) or prospectively, on data generated from 20–26 weeks' gestation (*n* = 3), upon the diagnosis of cCMV. In one woman (Case 6), CMV‐cfDNA analysis was performed at four timepoints: at 12 + 5 weeks (routine non‐invasive prenatal testing); 23 + 3 weeks (cCMV diagnosis); and 30 min and 12 h after termination of pregnancy (TOP) at 23 + 6 weeks.

**Results:**

CMV‐cfDNA was detectable in all cases. Mostly low levels of CMV‐cfDNA were observed in samples obtained at 11–13 weeks' gestation and consistently high levels of CMV‐cfDNA were present in samples obtained at cCMV diagnosis. In Case 6, the level of maternal CMV‐cfDNA decreased substantially in the samples tested after TOP, compared with samples obtained before TOP.

**Conclusions:**

Low levels of CMV‐cfDNA detected between 11 and 13 weeks may be a biomarker for severe fetal cCMV. CMV‐cfDNA analysis in the first trimester could be of added value in CMV screening, particularly for non‐primary maternal infections that cannot be identified using other methods. However, as CMV‐cfDNA is detectable in many pregnant women in the first trimester, further studies are needed to determine the predictive value of CMV‐cfDNA as a biomarker for the development of severe fetal cCMV. High levels of CMV‐cfDNA at fetal cCMV diagnosis and low levels directly after TOP suggest that the level of CMV‐cfDNA in maternal plasma may not necessarily reflect an active maternal infection, but could indicate a placental infection. © 2025 The Author(s). *Ultrasound in Obstetrics & Gynecology* published by John Wiley & Sons Ltd on behalf of International Society of Ultrasound in Obstetrics and Gynecology.

## INTRODUCTION

Screening for human cytomegalovirus (CMV) during pregnancy is not currently recommended by most national and international societies, because of the variation in clinical severity of a fetal infection and the lack of an internationally accepted therapy^1^. However, there is increasing evidence on the effectiveness of timely maternally administered valacyclovir to reduce maternal–fetal transmission of CMV after a maternal primary infection with onset in the periconceptional period or in the first trimester of pregnancy^2^, ^3^, ^4^, ^5^. Therefore, in their updated consensus recommendations for prenatal, neonatal and postnatal management of congenital cytomegalovirus (cCMV) infection that was published recently^6^, the European Congenital Cytomegalovirus Initiative (ECCI) advise that ‘in cases of a maternal primary infection in the periconceptional period or in the first trimester, oral valacyclovir should be administered as early as possible after the diagnosis and until the amniocentesis’. Considering these recommendations, the potential concerns of a large‐scale screening program should be balanced against the benefits of identifying pregnant women who are eligible for therapy. Currently, maternal CMV testing is based on serology, which is not available within the setting of a standard screening program in most countries. On the other hand, many countries offer non‐invasive prenatal testing (NIPT) for screening of fetal aneuploidies from 10 weeks' gestation onwards, either as a first‐tier test or as a second‐tier test. Previously, we and others have reported that, in whole‐genome cell‐free DNA (cfDNA) sequencing data, generated in the first trimester of pregnancy for the purpose of NIPT, CMV‐cfDNA fragments can be detected^7^, ^8^, ^9^, ^10^. These studies either focused on the technical potential to detect CMV‐cfDNA or concerned large‐scale studies without clinical follow‐up. The clinical relevance of the detection of such fragments, in terms of the activity status of the virus, risk on maternal–fetal transmission and potential eligibility for therapy, remains unclear. In the present study, for the first time, we have analyzed the CMV‐cfDNA profiles of nine pregnant women with symptomatic CMV‐infected fetuses at different timepoints during and after gestation, aiming to gain more insight on the relevance of the detection of CMV‐cfDNA fragments in maternal blood as a potential biomarker for severe cCMV infection.

## METHODS

### Study design and subjects

In this study, whole‐genome cfDNA sequencing data, generated according to NIPT procedures, were analyzed after the diagnosis of symptomatic cCMV by CMV quantitative polymerase chain reaction (qPCR) on amniotic fluid, fetal blood (cordocentesis) or neonatal urine. All pregnant women presenting at the Department of Obstetrics and Gynecology of the Radboud university medical center Nijmegen (RUMC), Nijmegen, The Netherlands (from June 2019 to July 2024 (*n* = 7)), and the Amsterdam University Medical Center (AUMC), Amsterdam, The Netherlands (from May 2023 to July 2024 (*n* = 2)), with a confirmed fetal or neonatal CMV infection were asked for their consent regarding retrospective reanalysis of their NIPT data, generated between 11 and 13 weeks' gestation, for the presence of CMV‐cfDNA fragments. Nine cases were included in the study, of which eight cases had a gestational age at the time of inclusion (i.e. when cCMV was established) between 20 + 4 and 34 + 1 weeks and one case was diagnosed neonatally. In The Netherlands, all pregnant women could opt for whole‐genome sequencing‐based NIPT, until April 2023 within the setting of the TRIDENT‐2 study^11^, from 11 weeks' gestation onwards, and since April 2023 within the setting of a routine first‐trimester screening program, from 10 weeks' gestation onwards. If first‐trimester NIPT had not been performed, women were asked for consent to perform prospective cfDNA sequencing, following standard NIPT procedures, for the purposes of this study only, without analysis for fetal aneuploidies. In addition to cfDNA data analysis, in most cases serology testing and CMV‐qPCR were performed on NIPT residual plasma.

The methods of this study met the criteria in relevant guidelines and regulations of the Declaration of Helsinki. Approval for the TRIDENT‐2 study^11^ was granted by the Dutch Ministry of Health, Welfare and Sport (license number: 1017420‐153371‐PG) and approved by the Medical Ethical Committees of the participating medical centers. The present study involved the retrospective analysis of data that had been generated previously, and, in some patients, an additional maternal blood sample was collected for the purposes of the study. These blood collections were scheduled at timepoints when a venal puncture had already been planned for diagnostic purposes. The Medical Ethical Committee of the RUMC Nijmegen judged the study to be exempt from assessment (file number: 2024‐17365).

### Strategy of routine diagnostic CMV testing

In general, serological methods (including avidity testing) and CMV‐qPCR testing were conducted and interpreted as described previously^10^.

For diagnostic CMV testing during pregnancy, maternal serology was performed to determine the presence of anti‐CMV‐IgM antibodies and anti‐CMV‐IgG antibodies. CMV‐qPCR on maternal blood samples obtained at diagnosis was also performed in some cases. In case of an intermediate or positive anti‐CMV‐IgM result, combined with anti‐CMV‐IgG‐positivity, anti‐CMV‐IgG‐avidity testing was carried out. To better estimate the onset of a maternal infection, if applicable, standard first‐trimester blood screening samples and/or NIPT residual plasma samples were traced and tested microbiologically to compare dynamics in antibodies.

If CMV‐qPCR of maternal blood was performed, viral load was determined and categorized as either (1) negative, (2) positive with a viral load of < 100 IU/mL (very low, non‐quantifiable but detectable load) or (3) positive with a viral load quantified in IU/mL (when viral load was > 100 IU/mL).

Fetal or neonatal infections were all established by CMV‐qPCR on amniotic fluid, fetal blood or neonatal urine; for these analyses, viral loads were not specified and data were classified as ‘positive’ or ‘negative’.

### CMV‐cfDNA analysis

CMV‐cfDNA analysis was performed as described previously^10^. Whole‐genome cfDNA sequencing data were analyzed for the presence of CMV‐cfDNA reads by aligning the non‐human reads to the CMV reference genome (human herpesvirus 5 strain Merlin genome (NCBI Refseq assembly accession GCF_000845245.1)). In the analysis, all reads that could be mapped according to previously specified criteria^10^ were considered.

## Results

Table 1 and Figure S1 provide an overview of the data obtained from CMV testing for all nine cases.

**Table 1:** Overview of cytomegalovirus (CMV) testing results for nine cases diagnosed with congenital CMV infection

		Routine diagnostic findings							
		Serology/qPCR on maternal blood				Results of CMV‐cfDNA analysis			
Case	GA at diagnostic serology testing (wks)	Sample at diagnosis (second/third trimester)	Routine first‐ trimester sample	qPCR on AF, FB or neonatal urine (GA)*	Pregnancy outcome	GA at CMV‐ cfDNA (wks)	CMV reads per 20 million total reads	Serology/ qPCR on leftover plasma	Onset of infection†
1	32 + 6	IgG‐pos‡ IgM‐pos‡ Avidity: high‡ qPCR: NT	IgG‐neg‡, §	Pos (AF) (34 + 1 wks)	TOP (35 + 0 wks)	11 + 1	0.6	IgG‐pos IgM‐pos Avidity: low qPCR: neg	Primary infection with onset periconceptionally or early in first trimester
2	21 + 1	IgG‐pos‡ IgM‐pos‡ Avidity: high‡ qPCR: pos (1000 IU/mL)	NA	Pos (AF) (21 + 1 wks)	TOP (22 + 3 wks)	12 + 2	4.7	IgG‐pos IgM‐pos Avidity: low qPCR: pos (425 IU/mL)	Primary infection with onset periconceptionally or early in first trimester
3	20 + 3	IgG‐pos IgM‐neg Avidity: NT qPCR: NT	IgG‐pos IgM‐neg Avidity: borderline	Pos (FB) (20 + 5 wks)	TOP (21 + 6 wks)	11 + 3	5.2	IgG‐pos IgM‐neg Avidity: NT qPCR: neg	Periconceptional primary infection or non‐primary infection
4	19 + 4	IgG‐pos‡ IgM‐neg‡ Avidity: NT qPCR: NT	NA	Pos (AF) (20 + 4 wks)	Unknown	11 + 0	1.4	IgG‐pos IgM‐neg Avidity: NT qPCR: pos (< 100 IU/mL)	Periconceptional primary infection or non‐primary infection
5	29 + 3	IgG‐pos IgM intermediate Avidity: high qPCR: NT	NT	Pos (AF) (29 + 3 wks)	TOP (32 + 2 wks)	11 + 4	1.8	NT	Most likely primary infection with onset in first trimester
6	22 + 3	IgG‐pos‡ IgM‐neg‡ Avidity: low‡ qPCR: pos (200 IU/mL)	NT (seronegative in previous pregnancy)	Pos (AF) (22 + 3 wks)	TOP (23 + 6 wks)	12 + 5 23 + 3 23 + 6¶ 23 + 6**	49.4 178.4 10.6 8.1	NT NT NT NT	Primary infection with onset periconceptionally or in first trimester
7	20 + 3	IgG‐pos IgM‐neg Avidity: NT qPCR: NT	NT	Pos (AF) (20 + 3 wks)	TOP (23 + 6 wks)	22 + 5	73.3	IgG‐pos IgM‐neg Avidity: NT qPCR: pos (1000 IU/mL)	Periconceptional primary infection or non‐primary infection
8	20 + 0	IgG‐pos IgM‐pos Avidity: high qPCR: pos (400 IU/mL)	NT	Pos (urine) (day 5 postpartum)	Liveborn neonate weighing 2258 g (37 + 3 wks)	20 + 0	142.7	NT	Uncertain; primary infection with onset periconceptionally or in first trimester or non‐primary infection
9	26 + 0	IgG‐pos IgM‐pos Avidity: high qPCR: NT	NT	Pos (AF) (26 + 0 wks)	TOP (28 + 2 wks)	17 + 2	124.1	NT	Uncertain; primary infection with onset periconceptionally or in first trimester or non‐primary infection

* Gestational age (GA) in weeks (wks) or days postpartum.

† Based on routine diagnostic serology data, combined with serology data on cell‐free DNA (cfDNA) leftover plasma.

‡ Test performed elsewhere.

§ Test was performed before pregnancy (no first‐trimester sample tested).

¶ Sample taken 30 min after termination of pregnancy (TOP) and placental birth.

** Sample taken 12 h after TOP and placental birth.

AF, amniotic fluid; FB, fetal blood; NA, not available; neg, negative; NT, not tested; pos, positive; qPCR, quantitative polymerase chain reaction.

### Case 1

#### Clinical description

Case 1 (gravida 2 para 1) had documented anti‐CMV‐IgG‐negative maternal serology results before pregnancy (conception via intracytoplasmic sperm injection) and was referred at 33 weeks' gestation because of fetal anomalies, possibly related to cCMV. Ultrasound imaging at RUMC revealed fetal growth restriction (FGR), abnormal Doppler results, including high velocity in the middle cerebral artery (MCA), microcephaly, small cerebellum (< 1^st^ percentile), intraventricular posterior synechiae, parenchymal calcifications, periventricular halo, small thalamus, abnormal opercularization and gyri formation, and enlarged pericerebral space. The placenta showed multiple calcifications.

Serology testing at 32 + 6 weeks' gestation (conducted in the referral center) revealed anti‐CMV‐IgG‐positivity and anti‐CMV‐IgM‐positivity, with high anti‐IgG‐avidity index. Amniocentesis performed at 34 + 1 weeks' gestation revealed a positive CMV‐qPCR, confirming a fetal CMV infection. The parents opted for late termination of pregnancy (TOP) at 35 + 0 weeks. A female fetus, weighing 1569 g, was delivered with jaundice and multiple petechiae, with a head circumference of 26 cm. Immunohistochemistry of the placenta showed CMV‐positivity.

#### CMV‐cfDNA analysis and additional serology/CMV‐qPCR testing

Routine first‐trimester NIPT had been performed at 11 + 1 weeks' gestation and 33 449 278 reads were generated. Following reanalysis of these data, one CMV read was detected (0.6 CMV reads per 20 million total reads). Serology testing on leftover plasma that was stored after NIPT showed anti‐CMV‐IgG‐positivity and anti‐CMV‐IgM‐positivity, with low avidity index, whereas CMV‐qPCR was negative.

#### Estimated timing of maternal infection

The serology results indicate a primary infection with onset in the periconceptional period or early in the first trimester of pregnancy.

### Case 2

#### Clinical description

Case 2 (gravida 2 para 1) was referred to RUMC at 19 + 6 weeks' gestation because fetal hyperechogenic bowel was noted on ultrasound and maternal serology was positive for anti‐CMV‐IgG and anti‐CMV‐IgM with high IgG‐avidity index. A leftover sample from routine first‐trimester blood screening was not available. At 20 + 2 weeks' gestation the fetus showed multiple congenital anomalies on ultrasound examination, including cerebellar hypoplasia, microcephaly, periventricular halo sign, hyperechogenic bowel, mild hyperechogenic kidneys and high peak velocity in the MCA, suggestive of cCMV. Amniocentesis at 21 + 1 weeks' gestation demonstrated a positive CMV‐qPCR, confirming a fetal CMV infection. CMV‐qPCR on maternal blood, drawn at the same time, showed a viral load of 1000 IU/mL. The patient opted for TOP at 22 + 3 weeks.

#### CMV‐cfDNA analysis and additional serology/CMV‐qPCR testing

Routine first‐trimester NIPT had been performed at 12 + 2 weeks' gestation and 21 058 200 reads were generated. Following reanalysis of these data, five CMV reads were detected (4.7 CMV reads per 20 million total reads). Serology testing on NIPT leftover plasma showed anti‐CMV‐IgG‐positivity and anti‐CMV‐IgM‐positivity, with low avidity index, and CMV‐qPCR was positive (425 IU/mL).

#### Estimated timing of maternal infection

The serology data suggest a primary infection with onset in the periconceptional period or early in the first trimester.

### Case 3

#### Clinical description

Case 3 (gravida 3 para 1) was referred to RUMC at 20 + 3 weeks' gestation because of fetal ascites. On ultrasound examination at 20 + 3 weeks' gestation, multiple signs suggestive of severe fetal anemia were observed, including cardiomegaly with a trace of tricuspid insufficiency, a thick edematous placenta and a high peak velocity in the MCA. Except for a head circumference below the 1^st^ percentile, no structural anomalies were seen. Maternal serology testing demonstrated anti‐CMV‐IgG‐positivity and anti‐CMV‐IgM‐negativity. Subsequent serology testing on a stored first‐trimester sample showed the same results with a borderline IgG‐avidity index. No conclusion could be drawn as to whether this concerned a periconceptional primary infection or a non‐primary infection. CMV‐qPCR on fetal blood (cordocentesis at 20 + 5 weeks' gestation) was positive, confirming a fetal infection. The parents opted for TOP at 21 + 6 weeks' gestation.

#### CMV‐cfDNA analysis and additional serology/CMV‐qPCR testing

Routine first‐trimester NIPT was performed at 11 + 3 weeks' gestation, with 19 257 548 reads generated after sequencing. Five CMV reads were detected following reanalysis of the sequencing data (5.2 CMV reads per 20 million total reads). Additional testing on NIPT leftover plasma showed anti‐CMV‐IgG‐positivity and anti‐CMV‐IgM‐negativity (avidity testing not performed). CMV‐qPCR was negative.

#### Estimated timing of maternal infection

Based on data from serology testing, a primary first‐trimester CMV infection as cause of the cCMV is unlikely, although a primary infection with onset in the periconceptional period cannot be ruled out.

### Case 4

#### Clinical description

Case 4 (gravida 1 para 0) was referred to RUMC at 19 + 4 weeks' gestation because of ultrasound findings that were suspicious for cCMV. Ultrasound examination at referral revealed severe FGR, ascites, cardiomegaly, high peak velocity in the MCA, reduced fetal movements and mild intracranial features. Other features included a slightly widened third ventricle, more prominent aspect of the lateral ventricles and walls, and a possible periventricular halo sign.

Serology testing (performed in the referral clinic at 19 + 4 weeks' gestation) had revealed anti‐CMV‐IgG‐positivity and anti‐CMV‐IgM‐negativity. A routine first‐trimester blood sample was not available for testing. CMV‐qPCR, performed on amniotic fluid obtained at 20 + 4 weeks' gestation, was positive, confirming a fetal infection. The patient returned to their home country for further care because of a language barrier. No further information is available on the outcome of this pregnancy.

#### CMV‐cfDNA analysis and additional serology/CMV‐qPCR testing

Routine first‐trimester NIPT was performed at 11 + 0 weeks' gestation and 28 453 686 reads were generated. Following reanalysis of these data, two CMV reads were detected (1.4 CMV reads per 20 million total reads). Serology testing on NIPT leftover plasma showed anti‐CMV‐IgG‐positivity and anti‐CMV‐IgM‐negativity (avidity testing was not performed). CMV‐qPCR was positive with a viral load < 100 IU/mL.

#### Estimated timing of maternal infection

Based on data from serology testing, a primary first‐trimester CMV infection as cause of cCMV is unlikely, although a primary infection with onset in the periconceptional period cannot be ruled out.

### Case 5

#### Clinical description

Case 5 (gravida 2 para 1) was referred at 29 + 3 weeks' gestation to the AUMC because of microcephaly. A targeted ultrasound scan revealed numerous anomalies suspicious for cCMV, including ventriculomegaly, calcifications in the brain, synechiae in the lateral ventricle of the brain and hyperechogenic bowel. Magnetic resonance imaging revealed polymicrogyria. Amniocentesis was performed and CMV‐qPCR on amniotic fluid was positive, confirming a fetal infection. At 29 + 3 weeks' gestation, maternal anti‐CMV‐IgM was borderline positive and anti‐CMV‐IgG was positive, with high anti‐CMV‐IgG‐avidity. Serology testing on a routine first‐trimester sample was not performed. Late TOP was performed on parental request at 32 + 2 weeks' gestation.

#### CMV‐cfDNA analysis and additional serology/CMV‐qPCR testing

Routine first‐trimester NIPT was carried out at 11 + 4 weeks' gestation and 34 088 038 reads were generated. Following reanalysis of these data, three CMV reads were detected (1.8 CMV reads per 20 million total reads). Additional testing on NIPT leftover plasma was not performed.

#### Estimated timing of maternal infection

The timing of CMV infection was unclear as first‐trimester serology data were lacking. A primary infection with onset early in the first trimester or periconceptional period, as well as a non‐primary infection, are possible causes for fetal infection.

### Case 6

#### Clinical description

Case 6 (gravida 2 para 1) was referred to RUMC at 22 + 3 weeks' gestation with several fetal anomalies on ultrasound, including FGR, hyperechogenic bowel, mega cisterna magna and severe microcephaly. Serology testing at 22 + 3 weeks' gestation showed anti‐CMV‐IgG‐positivity and anti‐CMV‐IgM‐negativity, with a low anti‐CMV‐IgG‐avidity index. CMV‐qPCR on maternal blood was positive (200 IU/mL). At 22 + 3 weeks' gestation, CMV‐qPCR on amniotic fluid was positive. Upon this finding, the couple decided for TOP. First‐trimester serology was not performed, but in a previous pregnancy the woman had tested seronegative for CMV.

#### CMV‐cfDNA analysis and additional serology/CMV‐qPCR testing

Routine first‐trimester NIPT was performed at 12 + 5 weeks' gestation, which generated 18 622 691 sequenced reads. After reanalysis of these data, 46 CMV‐cfDNA reads were detected (49.4 CMV reads per 20 million total reads). cfDNA analysis was additionally performed at 23 + 3 weeks' gestation, before TOP, and repeated 3 days later at 30 min and 12 h after TOP and placental birth. From the sample taken before TOP at 23 + 3 weeks' gestation, 19 165 507 reads could be sequenced, in which 171 CMV‐cfDNA reads were detected (178.4 CMV reads per 20 million total reads). In both samples drawn after TOP (at a hypothetical 23 + 6 weeks), nine CMV‐cfDNA reads were detected, from a total of 17 032 662 and 22 228 464 reads sequenced at 30 min (10.6 CMV reads per 20 million total reads) and 12 h (8.1 CMV reads per 20 million total reads) after placental birth, respectively. The viral loads of Case 6 at these different timepoints are shown in Figure S1.

#### Estimated timing of maternal infection

The serology results from the current and previous pregnancy suggest a primary infection with onset in the periconceptional period or in the first trimester of the current pregnancy. However, CMV seroconversion before the current pregnancy with a non‐primary CMV infection as the cause of cCMV cannot be ruled out.

### Case 7

#### Clinical description

Case 7 (gravida 1 para 0) was referred to RUMC at 20 + 3 weeks' gestation with ventriculomegaly and severe FGR. Ultrasound examination additionally showed a Blake's pouch cyst, a very small cerebellum, cardiomegaly, ascites, dilated hyperechogenic bowel and oligohydramnios. Amniocentesis for genetic testing and infection serology in maternal blood were performed. Maternal serology testing at 20 + 3 weeks' gestation showed anti‐CMV‐IgG‐positivity and anti‐CMV‐IgM‐negativity. A first‐trimester sample was not studied. Repeat ultrasound analysis, at 22 + 3 weeks' gestation, revealed hydrops fetalis, with signs of severe anemia. Subsequent CMV‐qPCR, performed on preserved supernatant of amniotic fluid, was positive for CMV. The couple opted for TOP.

#### CMV‐cfDNA analysis and additional serology/CMV‐qPCR testing

Blood was drawn for cfDNA testing at 22 + 5 weeks' gestation. In total, 33 542 346 reads were generated, in which 123 CMV‐cfDNA reads were detected (73.3 CMV reads per 20 million total reads). Serology testing on leftover plasma revealed anti‐CMV‐IgG‐positivity and anti‐CMV‐IgM‐negativity. CMV‐qPCR on the same sample was positive (1000 IU/mL).

#### Estimated timing of maternal infection

The results of serology testing suggest that a primary CMV infection as the cause of cCMV is unlikely, although a periconceptional primary infection cannot be ruled out.

### Case 8

#### Clinical description

Case 8 (gravida 3 para 1) was referred to RUMC at 20 + 0 weeks' gestation because of fetal hyperechogenic bowel. First‐trimester NIPT had not been performed, but NIPT was requested at referral to rule out fetal trisomy 21, which revealed a normal result. Serology testing at 20 + 0 weeks' gestation showed anti‐CMV‐IgG‐positivity and (weak) anti‐CMV‐IgM‐positivity with a high anti‐CMV‐IgG‐avidity index. CMV‐qPCR of maternal blood at that time was not reported, but in retrospect showed a (low) positive result (400 IU/mL). A first‐trimester sample was not available. The CMV‐testing results were interpreted as most probably related to an infection with onset more than 3 months previously. Amniotic fluid was not tested. Ultrasound examination at 31 + 3 weeks' gestation revealed a male fetus with bilateral hydronephrosis and dilated ureters. At 37 + 3 weeks, a neonate with low birth weight, disturbed renal function and microcephaly was born. On day 5 after delivery, a urine sample from the newborn tested positive for the presence of CMV and valganciclovir treatment was started for the duration of 6 months due to the severity of disease. Screening for hearing loss, eye involvement or other organ involvement, including central nervous system abnormalities, was negative. At 1 year of age, the infant showed normal development, without any signs of hearing loss or neurological complications.

#### CMV‐cfDNA analysis and additional serology/CMV‐qPCR testing

Blood was drawn for cfDNA testing at 20 + 0 weeks' gestation. In total, 19 344 234 reads were generated, in which 138 CMV‐cfDNA reads were detected (142.7 CMV reads per 20 million total reads).

#### Estimated timing of maternal infection

The timing of CMV infection based on serology data is uncertain. A primary infection with onset in the periconceptional period or early in the first trimester of pregnancy, as well as a non‐primary infection, are possible causes of the fetal infection.

### Case 9

#### Clinical description

Case 9 (gravida 3 para 1) was referred to AUMC at 26 + 0 weeks' gestation due to FGR and suspicion of a cCMV infection. Multiple fetal anomalies were seen on ultrasound, including microcephaly, a mild ventriculomegaly with periventricular flare, calcifications of the brain and a wide cisterna magna, hyperechogenic bowel and signs of myocarditis. Magnetic resonance imaging confirmed the ultrasound findings. Maternal serology was positive for anti‐CMV‐IgM and anti‐CMV‐IgG, with a high avidity index. CMV‐qPCR on amniotic fluid was positive. Upon parental request, a late TOP was carried out at 28 + 2 weeks' gestation.

#### CMV‐cfDNA analysis and additional serology/CMV‐qPCR testing

Blood for NIPT for the detection of fetal aneuploidies was drawn at 17 + 2 weeks' gestation and a total of 21 279 299 reads were generated. After reanalysis of these reads, 132 CMV‐cfDNA reads were detected (124.1 CMV reads per 20 million total reads). Additional testing on NIPT leftover plasma was not performed.

#### Estimated timing of maternal infection

The timing of the maternal CMV infection is uncertain as first‐trimester serology data are lacking. A primary infection with onset early in the first trimester of pregnancy or in the periconceptional period, as well as a non‐primary infection, are possible causes of cCMV.

### Summary of cases

In Cases 1–6, with cfDNA data between 11 and 13 weeks' gestation available, mostly low levels of CMV‐cfDNA were detected. In Cases 6–9, for which cfDNA analyses were carried out after cCMV confirmation, high levels of CMV‐cfDNA were encountered. In Case 6, the high level of CMV‐cfDNA at diagnosis sharply decreased shortly after TOP.

## DISCUSSION

In pregnant women diagnosed in the second trimester with a symptomatic CMV‐infected fetus, low levels of CMV‐cfDNA in whole‐genome NIPT cfDNA sequencing data between 11 and 13 weeks' gestation were detected retrospectively. As cases resulted from both maternal primary infections and non‐primary infections, this indicates a potential role for cfDNA analysis in the identification of first‐trimester active primary infections and non‐primary infections, with the latter not being identifiable by serological methods. When tested at the time of cCMV diagnosis, the CMV‐cfDNA level in maternal blood was higher, and this level substantially decreased very shortly after TOP in the single case tested (Case 6).

In all cases with first‐trimester data available, we detected CMV‐cfDNA, but the levels were consistently lower than at the time of cCMV diagnosis. As fetal infections with severe fetal sequelae are seen mostly in first‐trimester maternal–fetal transmissions^12^, we hypothesized that the low levels of CMV‐cfDNA in the first trimester are related to end‐phase activity of a maternal infection. A plausible explanation for the higher levels of CMV‐cfDNA observed in the cases with analyses after cCMV diagnosis may be the release of viral particles from the infected placenta. It is known that placental infection precedes transmission of the virus to the fetus^13^, ^14^, ^15^, ^16^, and the infected placenta is known to secrete viral particles into its environment^17^. Moreover, the highly fragmented genomes present in the plasma are considered non‐infectious^18^. Therefore, a test determining the presence of CMV‐cfDNA does not necessarily reflect the CMV status of the pregnant woman herself but may also be related to viral particles released from the placenta. This theory is also supported in our study by the single case with post‐TOP analyses (Case 6), which showed a sharp decrease in CMV‐cfDNA levels 30 min after placental birth, which was only 3 days after the sample obtained at diagnosis revealed a high level of CMV‐cfDNA.

During a 5‐year (for RUMC) and 1.5‐year (for AUMC) timeframe, we could investigate all women with symptomatic cCMV fetuses at the two academic centers involved in this study.

We detected CMV‐cfDNA in all cases, which were related to maternal primary infection as well as to non‐primary infection, for whom first‐trimester data were available. However, we could only analyze first‐trimester data from a limited number of cases, and we only detected (very) low levels of CMV‐cfDNA reads. Therefore, we do not claim that first‐trimester CMV‐cfDNA will always be detectable in women that develop symptomatic fetal cCMV, nor do we claim that low levels of CMV‐cfDNA will always lead to severe cCMV. Moreover, we only included severely affected cases that presented prenatally, whereas most symptomatic cCMV cases are only identified postnatally.

The ECCI recommends initial serology testing during pregnancy as soon as possible^6^. A recent study in France showed cost‐effectiveness for universal serology screening in conjunction with valacyclovir treatment^19^, but most European countries do not have a screening strategy in place. So far, the effectiveness of the administration of the antiviral agent, valacyclovir, has only been studied in women with a primary infection early in pregnancy^2^, ^3^, ^4^, ^5^. The effectiveness of valacyclovir in cases of non‐primary infection is still unknown, but one may argue that this is not influenced by the immune status of a pregnant woman. In their meta‐analysis, Kenneson and Cannon^20^ estimated the risk of maternal–fetal transmission after a non‐primary infection to be 1.4%, but this may be an underestimation. Whatever the exact risk may be, it is lower than that after a first‐trimester primary infection, which is estimated to be 36.8%^12^. Still, many severe cCMV infections result from non‐primary infections^21^. Unfortunately, with serology testing, non‐primary infections cannot be distinguished from harmless latent past infections and so, by screening serologically, only a proportion of the women whose fetuses are at risk for cCMV (i.e. those with a first‐trimester primary infection) can be identified and offered treatment. In the current study, this is indeed illustrated by at least Cases 3 and 4, which would not have been identified in the first trimester by serological methods as being at risk for cCMV.

As the sequence of events leading from maternal‐to‐placental‐to‐fetal infection takes between 6 and 8 weeks^16^, Amir *et al*.^5^ in 2023 proposed a revised protocol for valacyclovir treatment, with a maximum interval of 8 weeks between the onset of a primary infection in the first trimester and the initiation of treatment. Currently, NIPT is offered in most countries from week 10 onwards, either as a first‐tier or as a second‐tier screening test. With a reporting time of approximately 1 week, CMV‐cfDNA screening results could be available at 11–12 weeks' gestation. Even though screening earlier in pregnancy would enable the initiation of treatment sooner after the onset of an infection, results from CMV‐cfDNA screening using cfDNA sequencing data would still be in time to start treatment in case of an infection with onset after 3–4 weeks' gestation. Whether treatment with valacyclovir is a realistic strategy for all women with a positive CMV‐cfDNA result in the first trimester should be studied further in terms of its effectiveness, numbers needed to treat to prevent an affected child, maternal side effects and cost‐effectiveness. Alternatively, as with serology testing a primary infection can be identified with high sensitivity, CMV‐cfDNA screening might also be considered as second‐tier testing to identify active non‐primary infections in anti‐CMV‐IgG‐positive and anti‐CMV‐IgM‐negative women.

In conclusion, we have shown the potential for CMV‐cfDNA testing as a first‐trimester screening test, either as a first‐tier or second‐tier (after serology testing) test. Our study strongly suggests that the CMV‐cfDNA detected in maternal blood does not necessarily reflect a CMV infection in the mother herself, but viral particles may have also originated from the placenta. We urgently recommend the performance of large‐scale studies, with clinical follow‐up on both CMV‐cfDNA‐negative and CMV‐cfDNA‐positive women, to evaluate the clinical outcomes of their offspring (i.e. CMV‐infected or not, symptomatic or not). Future studies also need to consider the potential place of cfDNA screening within a CMV screening strategy, as a first‐ or second‐tier test, as well as costs and implementation issues.

## Supporting information


**Figure S1** Levels of cytomegalovirus (CMV) cell‐free DNA (cfDNA) from samples obtained in the first trimester (Cases 1–6), at congenital CMV diagnosis (Cases 6–9) or after termination of pregnancy (TOP) (Case 6). Circles denote individual cases whereas squares denote CMV‐cfDNA levels of Case 6 at all four timepoints.

## Data Availability

The data that support the findings of this study are available from the corresponding author upon reasonable request.
