# Supplementary figures and images for: Detection of human cytomegalovirus cell‐free DNA in pregnant women with symptomatically infected fetuses: proof‐of‐concept study
Source: Ultrasound Obstet Gynecol. 2025 Mar 3;65(4):470–7. doi: 10.1002/uog.29199 (PMC11961104; doi:10.1002/uog.29199)

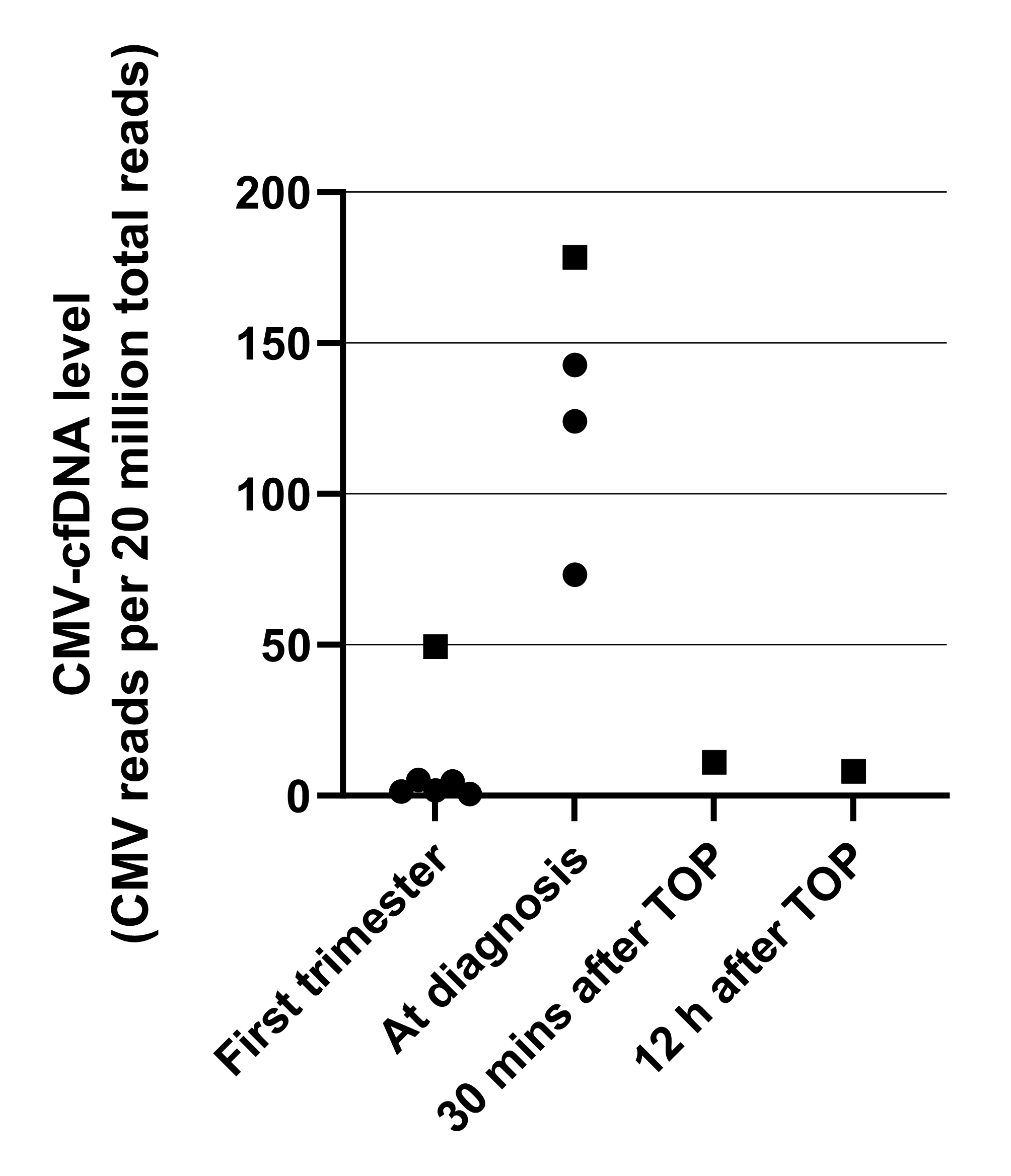

Supplement: Supplementary file 1 — Figure S1 Levels of cytomegalovirus (CMV) cell‐free DNA (cfDNA) from samples obtained in the first trimester (Cases 1–6), at congenital CMV diagnosis (Cases 6–9) or after termination of pregnancy (TOP) (Case 6). Circles denote individual cases whereas squares denote CMV‐cfDNA levels of Case 6 at all four timepoints. [file UOG-65-470-s001.png]
